# Retraction: MicroRNA-153 Inhibits Osteosarcoma Cells Proliferation and Invasion by Targeting TGF-β2

**DOI:** 10.1371/journal.pone.0269902

**Published:** 2022-06-08

**Authors:** The PLOS ONE Editors

Following the publication of this article [1], similarities were noted between this article and articles submitted by other research groups, including [2–10], of which one article [10] was previously retracted [11].

Similarities included the following figures which appear to partially overlap, despite being published in different articles and representing different conditions:

- Lanes 2–3 of the GAPDH panel in Fig 3D of [1], and lanes 1–2 of the GAPDH panel in Fig 4D of [6].

Although the corresponding author initially replied to acknowledge receipt of our message, *PLOS ONE* did not receive responses to the queries regarding these concerns by the end of the original deadline and extension.

The unresolved concerns call into question the validity and provenance of the reported results and the adherence of this article to the PLOS Authorship policy. Therefore, the *PLOS ONE* Editors retract this article [1].

All authors did not comment on the retraction decision, did not respond directly or could not be reached.
